# Immunostimulants in respiratory diseases: focus on Pidotimod

**DOI:** 10.1186/s40248-019-0195-2

**Published:** 2019-11-04

**Authors:** Francesca Puggioni, Magna Alves-Correia, Manar-Farouk Mohamed, Niccolò Stomeo, Riccardo Mager, Massimiliano Marinoni, Francesca Racca, Giovanni Paoletti, Gilda Varricchi, Veronica Giorgis, Giovanni Melioli, Giorgio Walter Canonica, Enrico Heffler

**Affiliations:** 1 Department of Biomedical Sciences, Humanitas University, Pieve Emanuele, MI Italy; 2 Personalized Medicine, Allergy and Asthma - Humanitas Clinical and Research Center – IRCCS, Via Alessandro Manzoni 56, 20089 Rozzano, MI Italy; 3 Central Hospital of Funchal, SESARAM, EPE, Madeira, Portugal; 4 Ain Shams University, Faculty of Medicine, Cairo, Egypt; 5 Department of Translational Medical Sciences and Center for Basic and Clinical Immunology Research, University of Naples Federico II, Naples, Italy

**Keywords:** Pidotimod, Immumostimulants, Respiratory diseases, Adaptive immunity, Innate immunity, Allergy, Asthma, Chronic obstructive lung disease

## Abstract

Usefulness of Pidotimod and its role as immunostimulant, has been discussed, we know, for several decades. Nevertheless, there is still much to know. Understanding its mechanisms and its potential usefulness in airway infections and its prevention, asthma both Th2 and non Th2 type, bronchiectasis, as adjuvant in vaccination and in allergen immunotherapy still remains to clearly unveil. The aim of this paper was to provide a useful updated review of the role of the main available immunostimulants, giving particular focus on Pidotimod use and its potentials utility in respiratory diseases. Pidotimod showed its usefulness in reducing need for antibiotics in airway infections, increasing the level of immunoglobulins (IgA, IgM, IgG) and T-lymphocyte subsets (CD3+, CD4+) endowed with immunomodulatory activity that affect both innate and adaptive immune responses. Higher expression of TLR2 and of HLA-DR molecules, induction of dendritic cell maturation and release of pro-inflammatory molecules, stimulation of T lymphocyte proliferation and differentiation toward a Th1 phenotype, as well as an increase of the phagocytosis have been demonstrated to be associated with Pidotimod in *in vitro* studies. All these activities are potentially useful for several respiratory conditions such as asthma, COPD, and recurrent respiratory tract infections.

## Introduction

Since the early 90’s more than a hundred papers have been published about Pidotimod, an immunostimulant drug. Due to its action mechanism and its potential, it has been used both in children and in adults, mostly for preventing respiratory tract infections, and asthma or chronic obstructive pulmonary disease exacerbations.

Despite the use of antibiotics and vaccines, the frequency of respiratory tract infections is still high and these infections disturb a wide range of patients, from children to elderly, particularly these two extremes due to the deficiency of their immune system: immaturity in the first case and “immunosenescence” in the second one. For that reason, immunostimulant drugs steadily increased in the past few years, getting nowadays more importance and visibility in preventing the onset and reducing the duration of airway infections [1].

Immunostimulants are a heterogeneous group of compounds that act non-specifically on the immune system by inducing its activation, either upregulating it or by favoring the activity of one of its components. By understanding deeply their biological function, they may be implemented in the clinical practice to shape the immune system favorably according to the different processes that want to be enhanced or hampered. According to these peculiar characteristics they have also been called “Biological Response Modifiers” (BRMs). Among this heterogeneous group of compounds which encompass both synthetic and naturally occurring substances, some of them deserve a brief discussion [2–31]. This review article will briefly summarize the most commonly used BRMs used in the treatment of respiratory diseases, and it will focus mainly on pidotimod and its current and potential clinical use.

*Bacterial Lysates* derive from a blend of bacterial cultures antigens throughout mechanical or chemical cell lysis. The selected bacteria include: *Streptococcus pneumoniae, Streptococcus pyogenes, Hemophilous influenzae, Legionella pneumophila, Moraxella catarrhalis, Mycoplasma pneumoniae, Chlamydia pneumonia, Streptococcus Aureus, Streptococcus viridans and Klebsiella pneumoniae.* These bacterial antigens have shown to directly upregulate both innate and adaptive immunity, by stimulating Toll-like receptors, inducing dendritic cells (DC) maturation, increasing phagocytic activity, and by activating interleukin 2 receptor (IL2R) on different lymphocytes subsets, inducing therefore cytokines synthesis and enhancing the production of IgA antibodies [2–4].

A Cochrane review by Del-Rio-Navarro et al. concluded that immunostimulants reduce the incidence of acute respiratory tract infections (ARTIs) by 40% in susceptible children, but the included trials quality was generally poor with a high level of statistical heterogeneity [5]; however, the subgroup analysis of bacterial lysates studies showed lower heterogeneity and displayed slightly better quality [5] and this was confirmed by a subsequent systematic review on OM-85 BV, a bacterial lysate [6]. Other beneficial effects of bacterial lysate in children are: reduction in the usage of antibiotics and duration of infectious episodes [7], faster improvement of the symptoms, shorter convalescence and decreased incidence of consequent infectious episodes when administered in children with sub-acute sinusitis [8].

In adults and elderly, polyvalent mechanical bacterial lysates (PMBL) have been shown to be superior respect to placebo and to polyvalent chemical bacterial lysates (PCML) in terms of reducing the number and the duration of infectious episodes, and reducing the need of antibiotic treatment in patients with recurrent respiratory tract infections (RRTI) [9]. Moreover, the high pressures used in PMBL production can eliminate some chemical contaminants with a lower degree of damage to bacterial antigens. PMBLs have been also shown to be beneficial in chronic obstructive pulmonary disease (COPD) patients by reducing the days of hospitalizations, increasing the interval between two exacerbations and reducing the number of days with fever; nonetheless they failed to reduce the number of exacerbations by 25% [10].

*Lactoferrin* is a natural iron-binding protein highly expressed by both epithelial cells in most exocrine secretions, and by neutrophils, which store the molecule in the secondary granules. It has been reported its functions as an alarmin-like molecule, promoting antigen presenting cells maturation and upregulation of proinflammatory cytokines synthesis [11], but also as a potent anti-inflammatory molecule on monocytes by triggering tolerogenic-like program during their differentiation into DC [12]; other reported functions of lactoferrin are: an inhibitory effect on eosinophils migration [13], interference with mast cells functions [14], and an effect on the immune Th1/Th2 balance [15].

The clinical effect of lactoferrin supplementation as a preventive measure for infectious diseases has been mainly investigated in newborn and preterm infants. Notably, an Italian study conducted by Manzoni et al. showed a significant decrease in the incidence of sepsis in very low birth weight infants (< 1500 g) who received 100 mg/d of bovine lactoferrin from birth until the 30 or 45 day of life [16].

*Resveratrol* is a natural polyphenolic compound contained in several plant species, such as peanuts, grapes and berries. It has been shown that resveratrol has an effect on a wide range of biological activities ranging from antimicrobial [17], neuroprotection [18] and anti-inflammatory [19]. Resveratrol’s molecular interactions still remain largely unknown, however it has been shown to act as an activator of sirtuin-1 (which is thought to play a role in activating Th17cells) [20], to modulate T-Reg/Th17 balance [21] and to downregulate pro-inflammatory cytokines production [22].

Notably, it has been demonstrated that resveratrol has a remarkable anti-inflammatory effect in COPD patients and its antimicrobial and immunomodulatory effects have been studied *in vivo* with a septicaemia infection model system induced by nontypeable *Haemophilus influenzae*(NTHi), showing a reduction in NTHi viability and a concomitant reduction in airway inflammation [23]. Furthermore, in a recent study conducted by Wu S et al*,* resveratrol reduced mortality, lung injury and cytokine levels in the lung tissue of *S. aureus* pneumonia in murine models [24]. Based on these studies, Resveratrol may be useful in the acute phase of pulmonary infections.

*Monophosphoryl lipid A* (MPLA) is a nontoxic, but still highly immunogenic derivative of lipid A, the biologically active part of lipopolysaccharide (LPS) endotoxin, and a Toll-like receptor 4 agonist. *In vitro* studies confirmed that MPLA is able to act, as its parent molecule LPS, as a potent adjuvant increasing the function of antigen presenting cells and an antibody response characterized by the production of IgG1 and IgG2 [25]. Because of its immunostimulant properties, MPLA has been used as adjuvant in malaria vaccine preparations [26], in hepatitis B surface antigen vaccine [27] and in papilloma virus vaccine [28]. Furthermore, it has been shown that MPLA promote allergen-induced immune deviation in favor of Th1 responses, confirming its beneficial role as an adjuvant in allergen immunotherapy [29, 30]. Interestingly, beside its role as adjuvant molecule, it has been demonstrated that MPLA administration before or after the induction of systemic bacterial infection results in an improved survival and an increased bacterial clearance [31, 32].

## Pidotimod

Pidotimod is a synthetic dipeptide molecule (3-l-pyroglutamyl-l-thiazolidine-4carboxilic acid) endowed with immunomodulatory activity that affects both innate and adaptive immune responses. Higher expression of Toll Like Receptors (TLR) 2 and of HLA-DR molecules, induction of DC maturation and release of pro-inflammatory molecules, stimulation of T lymphocyte proliferation and differentiation toward a Th1 phenotype, as well as an increase in the phagocytosis have been demonstrated to be associated with Pidotimod in *in vitro* studies [1, 33–35].

Several *in vitro* and *in vivo* studies on human and animals showed that pidotimod is able to ameliorate both innate and adaptive immunity, and enhances the immune system capabilities to fight RRTI, especially in children with increasing resistance to viral infections [36–40].

Studies in different areas have demonstrated the benefit of Pidotimod, including its use in hepatitis C, HPV genital infection, Henoch-Schönlein Purpura, nephrotic syndrome, and immunodepressed individuals such as children and elderly [1]. Considering its good safety profile, only one single case of potentially severe adverse event (new onset of Henoch-Schönlein Purpura) was reported to be associated to the intake of Pidotimod [41].

The common end-point of these studies is that Pidotimod has an immunomodulatory activity which is able both to improve the clinical conditions of patients and to enhance and stimulate their immunity cells functions acting on both adaptive and innate immunity [1].

The clinical efficacy of pidotimod in decreasing respiratory tract infections in paediatric age was assessed in multicenter randomized trials involving children with age ranged from 2 to 14 years old as regard fever duration, need to antibiotic treatment, hospitalization, school absence, recovery time and relapse rate. All these parameters were significantly reduced in the Pidotimod-treated groups, clarifying the supportive role of pidotimod in treatment of acute infections and its prophylactic role in prevention of RRTI [42, 43].

### Effect of pidotimod on innate immunity

It is well known that epithelial cells play a primary defensive role, not only providing a natural physical barrier but also being involved in innate and adaptive immune responses [1], by means of several mechanisms including the expression of TLRs on their surface. TLRs are pattern recognition receptors of molecules that are broadly shared by pathogens: these molecules are called pathogen-associated molecular patterns (PAMPs) [44, 45].

Recognition of PAMPs via TLRs results in the activation of NF-kB, which is a complex protein found in almost all animal cells and controls DNA transcription in response to variable stimuli, including bacterial and viral infections [46, 47]. Activated NF-Kb leads to stimulation of innate immune cells to release different cytokines and chemokines, eventually leading to internalization and phagocytosis of the attached pathogens [44, 45].

Carta et al designed an *in vitro* study to evaluate the immunomodulatory effects of pidotimod demonstrating that it is able to upregulate TLR2 expression, with significant increase in NF-kB protein expression and NF-kB nuclear translocation [48].

Pidotimod is also able to induce phenotypic and functional maturation of mucosal DC, which plays an important role in the cross talk between innate and adaptive immunity, as pidotimod upregulates the expression of HLA-DR and co-stimulatory surface markers CD83, CD88.

Pidotimod-induced DC maturation leads to release of pro-inflammatory mediators such as TNF-α leading to increase recruitment of inflammatory cells, activation of naïve T lymphocytes with proliferation and polarization toward Th1 phenotype. Besides its role on epithelial cells and DC, pidotimod also enhances cytotoxic activity of natural killer cells (NK) and phagocytic activity of neutrophils [49].

### Effect of Pidotimod on adaptive immunity

Recent immunological data concluded that functional disorder of Th1/Th2 cells could be strictly related to development of RRTI in children. *Zhou and Dai* proposed a study comparing the effect of Pidotimod to spleen aminopeptide (a cell immunity enhancer made of peptides and nucleotides extracted from spleen of healthy animals) on Th1/Th2 cytokines balance in children with RRTI [50]. Pidotimod was significantly associated with enhancement of secretion of IFN-γ and other Th1 cytokines such as IL12 through which Th1 mediates its inflammatory reactions and delayed hypersensitivity reactions more than spleen aminopeptide, on the same time it was able to down regulate IL4 secretion which is essential for Th2 activity [50].

It is appropriate to mention that Pidotimod effects on decreasing Th2 cytokines against increasing Th1 cytokines would not only enhance the immune system capabilities to fight infections, but it has also a protective role against the development of atopy; pidotimod also was found to down regulate CD30 expression on cells which is linked to Th2 cells [51].

A cohort randomized study held on children with Down syndrome (who are more prone to develop frequent respiratory tract infections), demonstrated that Pidotimod significantly upregulates genes involved in inflammation, chemotaxis and antimicrobial activity. Moreover, the ratio of flu-specific immunoglobulin G1/G3 (IgG1/IgG3) was skewed in Pidotimod treated individuals when it was given in association to seasonal virosomal adjuvanted influenza vaccine [52].

Moreover, increased production of nasopharyngeal and salivary secretory IgA (sIgA) in children with respiratory tract infections treated with Pidotimod has been demonstrated, letting suppose a possible direct effect of pidotimod on B lymphocytes [53].

Another study used nasal cytology to evaluate the ability of pidotimod to reduce nasal inflammation and improve children quality of life through a randomized controlled study involved 40 children with RRTI. Nasal cell motility, mucociliary clearance and the presence of supra nuclear-stria (SNS) were used as an indirect index of wellness of nasal ciliated cells: an improvement was seen in all these cytological features in the pidotimod treated group, with concomitant decreased frequency of respiratory tract infections and antibiotics consumption [54].

**Fig. 1:**
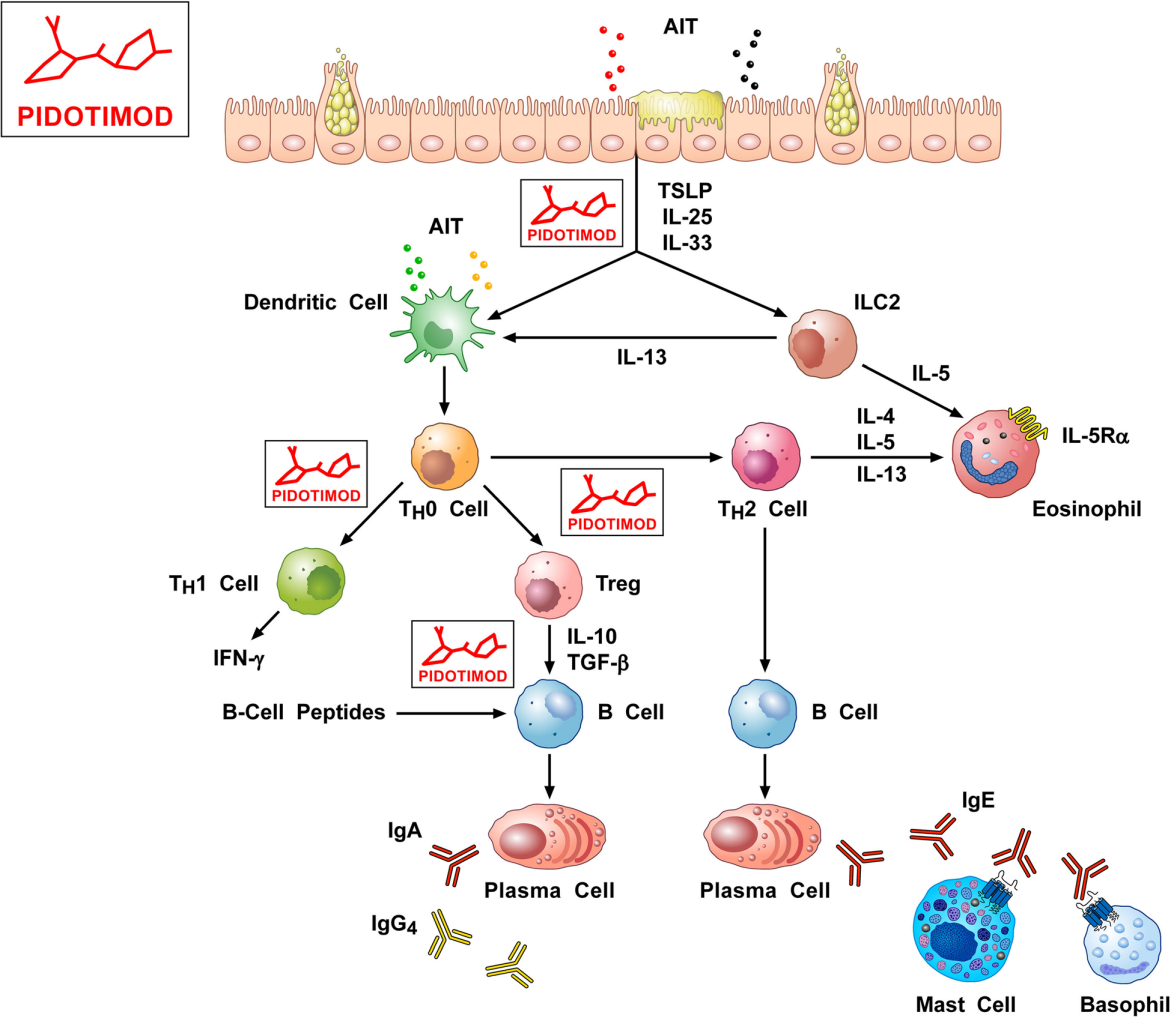
Immunological activities of Pidotimod

The main mechanisms of action of pidotimod, at both innate and adaptive immunity levels, are summarized in Fig. 1.

### Pharmacodynamics and safety of pidotimod

Pidotimod follows a first order pharmacokinetic when administrated through oral route, while parenteral route follows second order pharmacokinetics, with a half-life of 4 h with and an oral bio-availability of 42–44%. It is eliminated through the kidneys without being metabolized. Plasma clearance is 5 l × hE-1 with apparent distribution volume 30 l. Severeal types of oral route of the drug are available (tablets, sachets and vials) which are all bioequivalent [55].

Pidotimod has a good safety profile without increased frequency of reported side effects or autoimmune disorders in the pidotimod treated patients. Moreover, a study conducted on mice showed that pidotimod has no mutagenic effects either [56]. However, there is a single report in literature of Henoch-Schönlein Purpura associated with pidotimod therapy [41].

## Pidotimod in the treatment of airway diseases

Acute respiratory infections (ARIs) are still pandemics despite the introduction of new antibiotics and vaccines which contribute to reduce the risk of mortality and morbidity and they remain widespread and affect both young and elder people [1]. Furthermore, the socio-economic burden of ARIs remains high, considering the cost of symptomatic drugs, antibiotics, hospitalization and the indirect cost of absence from work or loss of school days [57].

### Viral infections

Most common causes of respiratory tract infections have viral origin especially human rhinoviruses (HRV), adenovirus, parainfluenza virus, respiratory syncytial virus (RSV), enterovirus, human metapneumovirus and coronavirus in addition to influenza viruses. [58, 59]. Viruses tend to cause direct invasion of the epithelial cell in the respiratory tract, the most common example of that is HRV which uses intercellular adhesion molecule (ICAM)-1 to gain access to human cells [60], with subsequent stimulation of production of pro-inflammatory cytokines including IL8, IL6, MCP-1 (a chemokine that recruits monocytes) leading to generation of inflammatory response, necrosis of host infected cells [61, 62], decreased ciliary clearance and increase mucous secretions. All these events will eventually cause subsequent obstruction of respiratory tract and impending drainage of secretions [63]. Impaired mucociliary escalator is a good media for bacteria proliferation and secondary infection (Particularly *streptococcus pneumoniae, Haemophilus influenzae*, and *Staphylococcus aureus*), causing increase in disease severity, mortality and morbidity with progression to unfavoured *sequela* such as pneumonia [64].

On the other hand, the host immune system tends to control viral infections via induction of apoptosis of infected cells using different mechanisms including: increasing TNF-alpha secretion, stimulation of natural killer (NK) cells to secrete perforin (making pores in the infected cells resulting in induction of cellular apoptosis) and stimulation of macrophages and neutrophils to produce reactive oxygen species causing oxidation of the infected host cell’s proteins, lipids and DNA leading to its death [65–67].

*Carta* et al.*,* held a study to assess the ability of pidotimod to induce cellular changes enhancing the host immune system to defend infections. This study has documented that pidotimod is able to upregulate TLR2, with no increase in ICAM or IL8 levels, thus playing a protective role in decreasing susceptibility to HRV infection and from neutrophil-mediated damage to the airway surface [48].

Another *in vitro* study aiming to evaluate the effect of pidotimod on immune system demonstrated that it is able to down-regulate MCP-1, which is a master regulator in inflammatory response associated with severe recurrent viral bronchiolitis in the children [68]. Moreover, it is able to upregulate NLRP12, which is a protective molecule against viral-induced abnormal inflammatory response [69].

### Lower respiratory tract infections

Lower respiratory tract infections (LRTIs), such as acute bronchitis and pneumonia, represent the most common cause of death from infectious diseases and the fourth overall most common cause of death worldwide. In 2016, it has been estimated that LRTIs have caused 2 million and 370 thousands deaths, among which more than 1 million where due to *S. pneumoniae* infection. Even if the incidence of LRTIs has decreased in the last decade in the pediatric population, LRTIs remain a leading, albeit preventable, cause of death among elderly patients [57].

Two different studies were planned to evaluate the *in vivo* immunomodulatory effect of pidotimod during acute community-acquired pneumonia (CAP) [70, 71]. One was held on children and the other was held on adults. In both studies, the patients were divided in two groups, one group receiving antibiotics only while the other antibiotics plus Pidotimod. Pidotimod induced the enforcement of the immune system stimulating some proteins such as lactoferrin, cathepsin G and myeloperoxidase, known to be endowed with potent antibacterial; similarly important is the finding that Pidotimod was associated with a reduced production of TNF-alpha, a proinflammatory cytokine whose excessive production is known as a negative prognostic factor in CAP. Finally, the finding that Pidotimod increased the expression of CD80 and CD86 on DC, confirms its role in triggering the adaptive immunity response [71]. Both studies indicated that pidotimod significantly increased the natural immune system response to an infectious stimulus via stimulation of DC maturation and increased TNFα and IL12 secretion. Pidotimod was found also to determine a long-term enhancement of the immune system activity, upregulating the expression of CCL3, CXCL1, CXCL2, IL-18,IL-1b, IL-6, IL-8, NFkB1, and NLRP3 genes involved in inflammation and chemotaxis, beside genes involved in antimicrobial activity (e.g., cAMP, lactoferrin, cathepsin G and Myloperoxidase), thus reducing the risk of early recurrences during CAP. [70, 71] These were pilot projects on small number of patients and with immunological (and not clinical) primary endpoints.

A recently published meta-analysis assessed a total of 29 RCTs consisting of 4,344 paediatric patients. Ten RCTs were published from Italy, Russia or Greece, and 19 RCTs were published by Chinese groups. Nonetheless, appropriate randomization methods were only used in 15 trials. Only one study had explicit allocation concealment. Since only eight RCTs were double-blind and placebo controlled, the evidence was not assessed as high quality. The meta-analysis indicates that treatment with Pidotimod resulted in a significant increase in the proportion of participants who had lower respiratory tract infections (RR 1.59; 95% CI 1.45–1.74, *p* < 0.00001) compared with the conventional treatment. Pidotimod could significantly decrease the duration of cough and fever. The number of patients using antibiotics was also remarkably decreased in the Pidotimod treatment group. Moreover, Pidotimod administration improved the levels of serum immunoglobulin (IgG, IgA, or IgM) and T-lymphocyte subtypes (CD3+, CD4+). Besides, Pidotimod administration did not increase the risk of adverse events of any cause (RR = 1.05, 95% CI 0.72–1.54, *p* = 0.80) [72].

### Chronic obstructive pulmonary disease (COPD)

COPD is a chronic inflammatory airway disease characterized by chronic fixed airway obstruction [73]. It affects more than 5 % of the population and is associated with high morbidity and mortality [74]. It is the third-ranked cause of death in the United States, causing more than 120,000 deceases annually [75], and it is one of the main causes of morbidity, clinician office visits, hospital admission and loss of working day in industrialized countries [76].

COPD results from complex interactions between environmental (mainly tobacco smoking and/or other pollutants) and molecular risk factors. Molecular risk factors for COPD include a plethora of gene polymorphisms, dysregulations of the antioxidants pool, metalloproteinase abnormalities and uncontrolled role of elastase. COPD exacerbations are triggered most often by respiratory viral infections, mainly rhinoviruses, even if bacterial component is present [77–79]. Moreover, exacerbations tend to be more severe and prolonged if caused by viral pathogens [80].

In terms of immunological response, aside from the inflammation, there is usually a neutrophilic recruitment; sputum eosinophilia can be also relevant, in particular during viral infections [81].

Being the reduction of exacerbation frequency and intensity paramount for the disease control, it appears fundamental to act on the immune system in order to prevent infections and to reach the optimum of care.

Due to its role of potentiating and regulating both the innate and acquired immune systems, Pidotimod appears to be promising also in COPD. In two Italian double-blind randomized trials Pidotimod showed significant reduction in infective COPD exacerbations in Pidotimod treated patients [82, 83]. This positive effect was not only present during the treatment period but also during the follow up, suggesting a protecting action of the drug and a possible use in cyclic regimen [83]. The long-lasting effect of Pidotimod was furtherly confirmed as it demonstrated to potentiate the immune response up to 5 weeks after infection [84].

Pidotimod was found effective also in a particularly susceptible population: elderly COPD patients; a population patients with severe COPD with different degrees of immunological signs of “immunosenescence” achieved a reduced number of exacerbations by the concomitant use of Pidotimod with flu vaccination compared to those treated with vaccine only [85].

In terms of bacterial exacerbations, another study showed that patients treated with Pidotimod plus amoxicillin/ clavulanic acid, had a faster remission of symptoms compared to those treated with antibiotics only [86]. Moreover, Pidotimod was seen able to increase the production of secretory IgA in patients with COPD, contributing in defining Pidotimod as a potential strategy to prevent infections [87].

### Asthma

Asthma phenotypes and endotypes based on inflammatory airway involvement have nowadays increasing medical interests thanks to the development of targeted therapies that aim to hamper directly the mechanism of the underlying pathologic processes [88]. At least two big groups of endotypes have been so far identified: an endotype characterized by Th2 cytokines overexpression (particularly IL5, IL4 and IL13) with concomitant airway eosinophilic inflammation, and a Th2-low inflammatory endotype in which neutrophils are the predominant inflammatory cells within the airways [89]. Th2-high endotypes of asthma can be further distinguished in allergic and non allergic but eosinophilic asthma [90]. The former is the result of an allergic reaction and more typically has a childhood onset, while the latter is the result of an eosinophilic inflammation toward an unknown trigger, it has typically an adult onset and is often associated to chronic rhinosinusitis with nasal polyposis and aspirin hypersensitivity [90]. All the above mentioned endotypes can present in a wide range of severity, and about 5–10% of all asthmatics are classified as affected by severe asthma as they require high dose inhaled corticosterpoids (ICS) plus a second controller and/or systemic glucocorticoids to prevent asthma from becoming ‘uncontrolled’ or which remains ‘uncontrolled’ despite this therapy [91]. One of the peculiar feature of severe asthma is the high frequency of exacerbations, often associated with respiratory tract infections, and accounting for the major fraction of the total heath-care related costs of asthma. [92] Moreover, a personalized approach to asthma cannot prescind from the diagnosis and treatment of comorbidities, in particular rhinitis and chronic rhinosinusitis with (CRSwNP) or without nasal polyps (CRSsNP). Recently, a cross-sectional study showed that CRS-related exacerbations is associated to lost productivity in asthmatics assessed as number of lost days of work or school [93]. Another common comorbidity of severe asthma is bronchiectasis [94] that it is estimated to be present in up to 25% of patients [95–97]; bronchiectasis is as an irreversible enlargement of the airways, which brings about a predisposition to develop recurrent and often severe lower respiratory tract infections, triggering asthma exacerbations [94]. Into this complex context in which patients with asthma, particularly if severe, are clearly more prone to develop exacerbations linked to upper and/or lower airway infections, an immunostimulatory approach with Pidotimod may be beneficial.

Pre-clinical evidence are already available: in a study investigating the ability of Pidotimod to affect in vitro the phenotype and cytokine profile of blood cells in relation to atopic asthma showed that it was able to down-regulate the expression of CD30 on mononuclear cells isolated from both atopic asthmatic children and healthy controls [51]. Because CD30 has been associated with Th-2 cells, this observation supports the possibility of Pidotimod being able to affect the Th-1/Th-2 balance in atopic asthma.

T2-low asthma endotype patients tend to not react to targeted therapies for pathologic type 2 inflammation, such as glucocorticosteroids. Some, recent studies support the theory that NK cell cytotoxic function is disabled in part by steroids in severe asthma. These NK cells express specialized pro-resolving mediators (SPMs) that are pivotal signals for the resolution of tissue inflammation; nevertheless, SPM abundance or signalling receptors are disrupted in chronic inflammatory disease. SPM receptors and SPMs can in some instances counter the deleterious effects of corticosteroids on the effector function of NK cells. Together, these findings suggest that some severe asthma, patients already refractory to the beneficial actions of corticosteroids, may be harmed by the steroids, increasing susceptibility to viral infection and asthma exacerbations. Thus, leaving an unveiled indication for the use of Pidotimod in these patients once its acts in the prevention of viral infections and asthma exacerbations [98].

### Allergy

In Europe, approximately 23% of the population is affected by allergic rhinitis (AR) [99] that is frequently accompanied by allergic asthma (AA), the prevalence of which increases from less than 2% in individuals without AR to 10–40% in those with AR; this combination of upper and lower respiratory symptoms increases the overall impact on the patient. There is now considerable evidence that the symptoms of AR and AA negatively affect patients’ health related quality of life [100–102].

Allergen immunotherapy (AIT) is nowadays the only treatment specifically targeting the allergic inflammatory pathways and it represents a prototype of personalized medicine approach to respiratory disease patients [88]. AIT is highly effective and may induce long-term remission of symptoms. However, ongoing problems include the duration of treatment, the safety of immunotherapy, its cost and accessibility [103].

Adjuvants have the potential to modify the pharmacological and immunological effects of allergen vaccines. They may modulate allergen delivery, act as a depot, stimulate immune responses or limit antibody responses in order to reduce unwanted side effects.

Adjuvants may be used in combination with potentially cumulative effects. Therefore, since Pidotimod action pathways include an effect against TSLP and TNF-α action (Fig. 1), it could open an interesting and unexplored option of adjuvant treatment in allergen immunotherapy depending on the choose route of administration.

#### Potential use of Pidotimod as adjuvant in vaccination

A recent study, used Pidotimod in chickens vaccinated with Newcastle disease virus vaccine revealing that Pidotimod could significantly promote growth performance, lymphocyte proliferation, enhance serum antibody titer, CD4/CD8 cell ratios and improve serum IL-2 and IFN-γ concentrations, indicating that it significantly improves the immune efficacy of Newcastle disease vaccine, once again reinforcing the role of immunostimulant that already is well established in previous literature findings [104].

## Conclusions

In the last years several immunostimulants of natural or synthetic origins, and working with different mechanisms, have been created for the prevention of RRTIs [1]. Clinical experiences show that there is good evidence about the role of immunostimulants as adjuvant treatment of respiratory diseases in children, while far less exists for adults. Among the currently available immunostimulants, Pidotimod have the potential characteristics to be one of the most effective adjuvant strategies for respiratory diseases.

Further high-quality, large-scale randomized controlled studies and real-life experiences are essential to provide confirmatory evidence.

## Data Availability

Not applicable.

## Abbreviations

AA: Allergic asthma
AIT: Allergen immunotherapy
AR: Allergic rhinitis
ARIs: Acute respiratory infections
BRMs: Biological Response Modifiers
CAP: Community-acquired pneumonia
COPD: Chronic obstructive pulmonary disease
CRSsNP: Chronic rhinosinusitis without nasal polyps
CRSwNP: Chronic rhinosinusitis with nasal polyps
DC: Dendritic cells
HRV: Human rhinoviruses
ICAM: Intercellular adhesion molecule
IL2R: Interleukin-2 receptor
LPS: Lipopolysaccharide
LRTIs: Lower respiratory tract infections
MPLA: Monophosphoryl lipid A
NK: Natural killer
NTHi: Nontypeable Hemophilus influenzae
PAMPs: Pathogen-associated molecular patterns
PCML: Polyvalent chemical bacterial lysates
PMBL: Polyvalent mechanical bacterial lysates
RRTI: Recurrent respiratory tract infections
SNS: Supranuclear stria
SPM: Specialized pro-resolving mediators
TLR: Toll like Receptor
URTI: Upper respiratory tract infection
